# Mutational status predicts the risk of thromboembolic events in lung adenocarcinoma

**DOI:** 10.1186/s40248-017-0097-0

**Published:** 2017-07-03

**Authors:** Elsa Davidsson, Nicola Murgia, Cristian Ortiz-Villalón, Emil Wiklundh, Magnus Sköld, Karl Gustav Kölbeck, Giovanni Ferrara

**Affiliations:** 1 Department of Respiratory Medicine and Allergy, Karolinska University Hospital, Stockholm, Sweden; 2 Section of Occupational Medicine, Respiratory Diseases and Toxicology, University of Perugia, Perugia, Italy; 3 Department of Pathology, Karolinska University Hospital, Stockholm, Sweden; 4 Oncology-Pathology Unit, Karolinska Institutet, Stockholm, Sweden; 5 Department of Medicine Solna, Karolinska Institutet, Karolinska University Hospital, Stockholm, 17176 Sweden

**Keywords:** Non-small cell lung cancer, Thromboembolism, Mutation, Tyrosine kinase inhibitors, Precision medicine

## Abstract

**Background:**

Precision medicine promises to improve prognosis of patients affected by untreatable diseases. Patients with lung cancer (especially lung adenocarcinoma) bear an increased risk of VTE. Mutations in the EGFR and rearrangement in the ALK genes identify specific subgroups of patients. Aim of this study was to investigate the role of epidermal growth factor receptor (EGFR) and anaplastic lymphoma kinase (ALK) mutational status on the risk of venous thromboembolism (VTE) in lung adenocarcinoma.

**Methods:**

A retrospective longitudinal design was used. Patients with lung adenocarcinoma diagnosed and undergoing a mutational analysis at the Karolinska University Hospital, Stockholm, Sweden between January 2009 and September 2015 were divided in three subgroups based on their mutational status (EGFR-, ALK-mutated, unexposed group). Event-free time for VTE was assessed using Cox regression analysis based on mutation status and treatment received.

**Results:**

Three hundred-ten patients were included. A VTE occurred in 70 (22.6%) patients. Mutation of EGFR was associated with a decreased risk of VTE (HR 0.46, 95% CI 0.23–0.94). Treatment with tyrosine kinase inhibitors (TKI) reduced the risk of VTE compared to other treatment strategies not including TKI (HR 0.42, 95% CI 0.29–0.79).

**Conclusions:**

Our study suggests that patients with lung adenocarcinoma bearing a EGFR-mutation have a decreased risk of VTE compared with patients with other forms of lung adenocarcinoma. Targeted therapy with TKI alone or in combination with other treatments seems to reduce the risk of VTE compared to other treatments not including TKI.

## Background

Precision medicine is rapidly changing the way chronic and untreatable diseases are managed [1]; it relies on the possibility to characterize patients at a molecular level and to offer them the best treatment at the right time based on the intrinsic mechanisms of their disease [2].

Lung cancer is the leading cause of cancer-related death for both men and women [3, 4]. In Sweden, the 5-year survival is still 13.6% among men and 19.4% among women [4]. Lung adenocarcinoma is the most common type of lung cancer in non-smokers, as well as in smokers and ex-smokers [2, 3]. Molecular analyses to detect epidermal growth factor receptor (EGFR) mutations and anaplastic lymphoma kinase (ALK) rearrangement identify specific subgroups of patients [5, 6], often younger and non-smokers compared to other forms of adenocarcinoma [7–9]. Targeted treatments with tyrosine kinase inhibitors (TKI) have become the first line therapy for patients with advanced lung adenocarcinoma bearing EGFR mutations or ALK rearrangement, thanks to the improved clinical response and tolerability profile of these drugs compared to standard chemotherapy [10, 11]. By inhibiting the signaling of mutated receptor tyrosine kinases, TKI target the pathogenic mechanisms of the disease [12, 13]. Unfortunately, a main problem is the development of resistance to TKI during treatment [14].

The association between cancer and thromboembolism was first described in 1865 by Armand Trousseau, and the term Trousseau’s syndrome is still used to describe a state of hypercoagulability in cancer patients [15]. Thromboembolic disease is a common cause of death among these patients [15, 16], and those with lung cancer are among the cancer groups with the highest risk of deep venous thrombosis (DVT) or venous thromboembolism (VTE) [17]. Lung adenocarcinoma is associated with a higher risk of VTE compared with other types of lung cancer [17–19]. It is still unknown how the EGFR and ALK mutational profile affects the risk of DVT/VTE in lung adenocarcinoma. The aim of this study was therefore to evaluate the occurrence of DVT/VTE in patients with EGFR- and ALK-mutated forms of lung adenocarcinoma compared with non-mutated cases. Furthermore, we evaluated the potential impact of different treatment strategies on the risk of VTE.

## Methods

### Patients

In the Stockholm County (approximately 2.2 million inhabitants), Sweden, diagnostic work-up and treatment of lung cancer is centralized at the Karolinska University Hospital in Stockholm. A list of all the mutational analysis tests for lung adenocarcinoma performed between January 1, 2009 and September 15, 2015 at the Department of Pathology of the Karolinska University Hospital was retrospectively reviewed in order to include all the patients bearing one of the EGFR mutations (EGFR group) or the ALK rearrangement (ALK group); the unexposed group included patients who tested negative for any mutation (EGFR, KRAS, ROS-1, BRAF mutations or ALK rearrangement) randomly extracted from the same list. Patients with other forms of lung cancer than adenocarcinoma were excluded.

Mutational status was tested for all the patients with Cobas® EGFR Mutation test kit (CE-IVD) (Roche Molecular Systems, Inc., Branchburg, NJ, USA) for the EGFR mutations and with immunohistochemistry (Ventana Medical Systems, Inc, Tucson, AZ, USA) and fluorescence in situ hybridization (FISH) (Abbott Molecular, Des Plaines, IL, USA) for the ALK rearrangement.

### Data collection

Data were retrospectively collected from the patients’ electronic medical records with coded anonymous IDs. The following variables were collected for each patient: Age, gender, survival and co-morbidity (diabetes, hypertension); smoking status: Never-smoker (smoked less than 6 months), ex-smoker (have not smoked in the last 6 months) and current smoker; tumor-related variables: Stage according to TNM classification, mutational status and main treatments; target events: VTE or DVT, time from diagnosis, on-going anti-coagulant treatment (preventive dosage) and time from death; performance status 0–4 (assessed according to World Health Organization/Eastern Cooperative Oncology Group) [20]. Target events were all radiologically confirmed with computed tomography (CT) with intravenous contrast (VTE) or ultrasound (DVT).

### Statistical analysis

Descriptive analyses include mean and standard deviation of the mean (SD) for the continuous variables and proportions of the total for the categorical variables. Subgroup analyses were performed for each of the three study groups. For comparison of categorical variables *x* ^2^-test was used. Incidence rates and their confidence intervals were calculated for all the categorical variables, grouping treatment options in four categories (TKI alone, conventional chemotherapy alone, other treatments in combination with TKI, other treatments without TKI).

Event-free time for target events was assessed using Cox regression analysis according to mutation status and treatment, adjusting for other relevant factors (age, gender, smoking status, treatment with a preventive dose of low molecular weight heparin and stage). The choice of the covariates to adjust for was based on statistical relevance by Kaplan-Meier curves for the single covariate (e.g., age and gender) or by a biological relevance (e.g., anti-coagulant treatment). Two different Cox regression models, one with mutational status and one considering treatment options, were performed to avoid the expected excess of collinearity between these two variables. In these models, age was considered as a categorical variable with cut-off at 66 years as median age of the population. Patients with a target event at the time of diagnosis were excluded from the Cox analysis. Level of significance was set at *p* < 0.05. Statistics were performed with SPSS 23 edition (IBM Corporation, Chicago, Illinois).

### Ethics

The main investigator (GF) contacted the Regional Ethical Committee in the Stockholm County to discuss this study. The Committee considered this study as a quality control and quality improving process. As such, no ethical permission is necessary according to the Swedish law. The study was approved by the direction of the Department of Respiratory Medicine and Allergy, Karolinska University Hospital, Stockholm, Sweden, as a quality control and quality improving process at the Department.

## Results

One thousand thirteen patients with lung adenocarcinoma underwent a mutational analysis test during the study period at the Karolinska University Hospital; among them, 104 (10.3%) tested positive for a EGFR mutation, 52 (5.1%) had an ALK translocation. The unexposed group included 154 (15.2%) patients with a negative test result for any mutation. Demographics and clinical features of the enrolled population are showed in Table 1. In the entire study population, 159 (51.3%) patients were in stage IV at diagnosis; more patients were diagnosed in stage IV in the mutated groups compared to the unexposed group (*p* > 0.001, Table 1). Performance status 0 was assessed in 203 (65.5%) patients at diagnosis, meaning that most of the patients were fully active at that time; no statistical difference was observed among the groups (Table 1). As expected, there was a difference in smoking habits: both of the mutated groups had a higher number of never-smokers (*n* = 45, 43.3% and *n* = 26, 50% respectively in the EGFR- and ALK-group compared to *n* = 15, 9.7% in the unexposed group, *p* < 0.001) and the unexposed group had more current smokers (*n* = 52, 33.8% compared to *n* = 9, 8.7% and *n* = 6, 11.5% in the EGFR- and ALK-group, respectively, *p* < 0.001).

**Table 1:** Demographics and clinical features of patients at time of diagnosis

Variable	EGFR (*n* = 104)	ALK (*n* = 52)	Unexposed group (*n* = 154)	Total (*n* = 310)	*p*-value
Age mean ± SD	66.9 ± 12.0	57.6 ± 11.9	67.4 ± 9.4	65.6 ± 11.3	
Gender:					NS
Female	68 (65.4%)	32 (61.5%)	86 (55.8%)	186 (60.0%)	
Male	36 (34.6%)	20 (38.5%)	68 (44.2%)	124 (40.0%)	
Stage:					*<0.00*
I	18 (17.3%)	4 (7.7%)	41 (26.6%)	63 (20.3%)	1
II	8 (7.7%)	1 (1.9%)	24 (15.6%)	33 (10.6%)	
III	7 (6.7%)	13 (25.0%)	35 (22.7%)	55 (17.7%)	
IV	71 (68.3%)	34 (65.4%)	54 (35.1%)	159 (51.3%)	
Performance status:					NS
0	67 (64.4%)	37 (71.1%)	99 (64.3%)	203 (65.5%)	
1	33 (31.7%)	12 (23.1%)	46 (29.9%)	91 (29.4%)	
2	2 (1.9%)	2 (3.8%)	5 (3.2%)	9 (2.9%)	
3	2 (1.9%)	1 (1.9%)	4 (2.6%)	7 (2.3%)	
Smoking status:					*<0.00*
Never-smoker	45 (43.3%)	26 (50.0%)	15 (9.7%)	86 (27.7%)	1
Ex-smoker	50 (48.1%)	20 (38.5%)	87 (56.5%)	157 (50.6%)	
Smoker	9 (8.7%)	6 (11.5%)	52 (33.8%)	67 (21.6%)	
Diabetes	7 (6.7%)	2 (3.8%)	15 (9.7%)	24 (7.7%)	NS
Hypertension	31 (29.8%)	11 (21.2%)	64 (41.6%)	106 (34.2%)	*0.014*
AC before the event	7 (6.7%)	9 (17.3%)	19 (12.3%)	35 (11.3%)	NS

*Abbreviations*: *EGFR* epidermal growth factor receptor, *ALK* anaplastic lymphoma kinase, *SD* standard deviation, *AC* anticoagulants, preventive dose for venous thromboembolism

In the whole study population, a target event occurred in 70 (22.6%) patients: the course of the disease was complicated in 17 (5.4%) patients by a DVT, in 51 by a VTE (16.5%) and in two (0.7%) patients by both DVT and VTE.

Eight (7.7%) patients in the EGFR-group, four (7.7%) in the ALK-group and five (3.2%) in the unexposed group had a target event already at diagnosis. These 17 patients were therefore excluded, leaving 293 patients (94.5% of the total study population) and 53 target events (75.7% of all the events) for the Cox regression analysis. The follow up time ranged between 10 and 3160 person/days. The sum of person/days in the whole population was 232,060. Overall incidence rate was 0.23 × 1000 person/days (95%CI 0.17–0.29). Frequencies of target events in relation to patients’ demographics and characteristics at diagnosis are presented in Table 2.

**Table 2:** Incidence rate of target events (venous thromboembolism, deep venous thrombosis)

Variable	Target event (n)	Incidence rate (95% CI)
Mutation status:		
EGFR+	15	0.21 (0.10–0.32)
ALK+	13	0.27 (0.13–0.42)
Unexposed group	25	0.22 (013–0.31)
Gender:		
Female	31	0.21 (0.14–0.29)
Male	22	0.26 (0.15–0.37)
Age		
< 66 years	38	0.33 (0.22–0.43)
> 66 years	15	0.13 (0.06–0.19)
Stadium		
I	4	0.05 (0.01–0.10)
II	6	0.21 (0.04–0.37)
III	9	0.18 (0.06–0.30)
IV	34	0.46 (0.30–0.61)
Smoking status:		
Never smoker	17	0.26 (0.14–0.38)
Ex-smoker	14	0.18 (0.11–0.26)
Smoker	22	0.26 (0.14–0.38)
Hypertension	12	0.14 (0.06–0.22)
Diabetes	0	-
AC before the event	7	0.24 (0.06–0.42)
Treatment options:		
CTP alone	30	1.00 (0.55–1.45)
Other treatment without TKI	4	0.11 (0.05–0.17)
Other treatment with TKI		0.25 (0.13–0.38)
TKI alone	19	0.15 (0.04–0.29)

Incidence rate (x1000 person/days) in relation to mutation status, baseline clinical features, and treatment options

*Abbreviations*: *EGFR+* patients bearing a mutation in the epidermal growth factor receptor (EGFR), *ALK+* patients bearing a rearrangement of the anaplastic lymphoma kinase (ALK), *AC* anticoagulants, preventive dose for venous thromboembolism, *CTP* standard chemotherapy, *TKI* tyrosine kinase inhibitors

Forty-three patients (14.7% out of the 293 patients included in the Cox analysis, 37 patients in the EGFR-group, four in the ALK-group and two in the unexposed group) received treatment with TKI only. Totally 58 (19.8% of 293) patients, including two patients not bearing a EGFR- or ALK-mutation, received TKI as first line treatment. Fifty-six (38.8%) and 40 (27.8%) out of the 144 patients with confirmed EGFR- or ALK-mutation were treated with TKI as first or second line treatment, respectively.

Forty-two patients (14.3% out of the 293 patients included in the Cox analysis, five patients in the EGFR group, eight in the ALK group and 29 in the unexposed groups) received treatment with chemotherapy only. Table 3 shows data about treatment-combinations in the study population divided according mutational status.

**Table 3:** Frequency of prescribed treatment and treatment combinations in the study population, grouped by mutational status

Treatment	EGFR (*n* = 96)	ALK (*n* = 48)	Unexposed group (*n* = 149)	Total (*n* = 293)
TKI	37 (38.5%)	4 (8.3%)	2 (1.3%)	43 (14.7%)
TKI + surgery	4 (4.2%)	1 (2.1%)	0 (0%)	5 (1.7%)
CTP	5 (5.2%)	8 (16.7%)	29 (19.5%)	42 (14.3%)
CTP + surgery	0 (0%)	2 (4.2%)	34 (22.8%)	36 (12.3%)
CTP + TKI	26 (27.1%)	24 (50%)	0 (0%)	50 (17%)
RTP	2 (2.1%)	1 (2.1%)	13 (8.7%)	16 (5.5%)
Surgery	10 (10.4%)	3 (6.3%)	30 (20.1%)	43 (14.7%)
Combination (TKI, CTP, RTP and/or surgery)	7 (7.3%)	3 (6.3%)	30 (20.1%)	40 (13.7%)
No treatment	5 (5.2%)	2 (4.2%)	11 (7.4%)	18 (6.1%)

*Abbreviations*: *EGFR* epidermal growth factor receptor, *ALK* anaplastic lymphoma kinase, *TKI* tyrosine kinase inhibitors, *CTP* chemotherapy, *RTP* radiotherapy

The Cox regression analysis identified variables affecting the risk of DVT/VTE: Mutational status was associated with a lower risk of target events for the EGFR group, as well as age >66, while stage IV was associated with an increased risk (Table 4). Adjusting also for smoking status did not alter the results (data not shown).

**Table 4:** Cox regression analysis for risk of thromboembolic disease in lung adenocarcinoma

Variable	HR (95% CI)
Gender	
Male	1
Female	0.83 (0.47–1.45)
Age	
< 66	1
> 66	*0.44 (0.23–0.82)*
Stadium	
I	1
II	3.10 (0.86–11.25)
III	3.11 (0.94–10.32)
IV	*8.70 (2.90–26.17)*
AC before the target event	
NO	1
YES	1,10 (0.49–2.50)
Mutational status	
Non exposed	1
EGFR+	*0.46 (0.23–0.94)*
ALK+	0.61 (0.29–1.29)

Significant covariates are shown. Adjusted for gender

*HR* hazard ratio, *95% CI* 95% confidence interval, *NS* not significant, *AC* anticoagulants, preventive dose for venous thromboembolism, *EGFR+* patients bearing a mutation in the epidermal growth factor receptor (EGFR), *ALK+* patients bearing a rearrangement of the anaplastic lymphoma kinase (ALK)

**Fig. 1:**
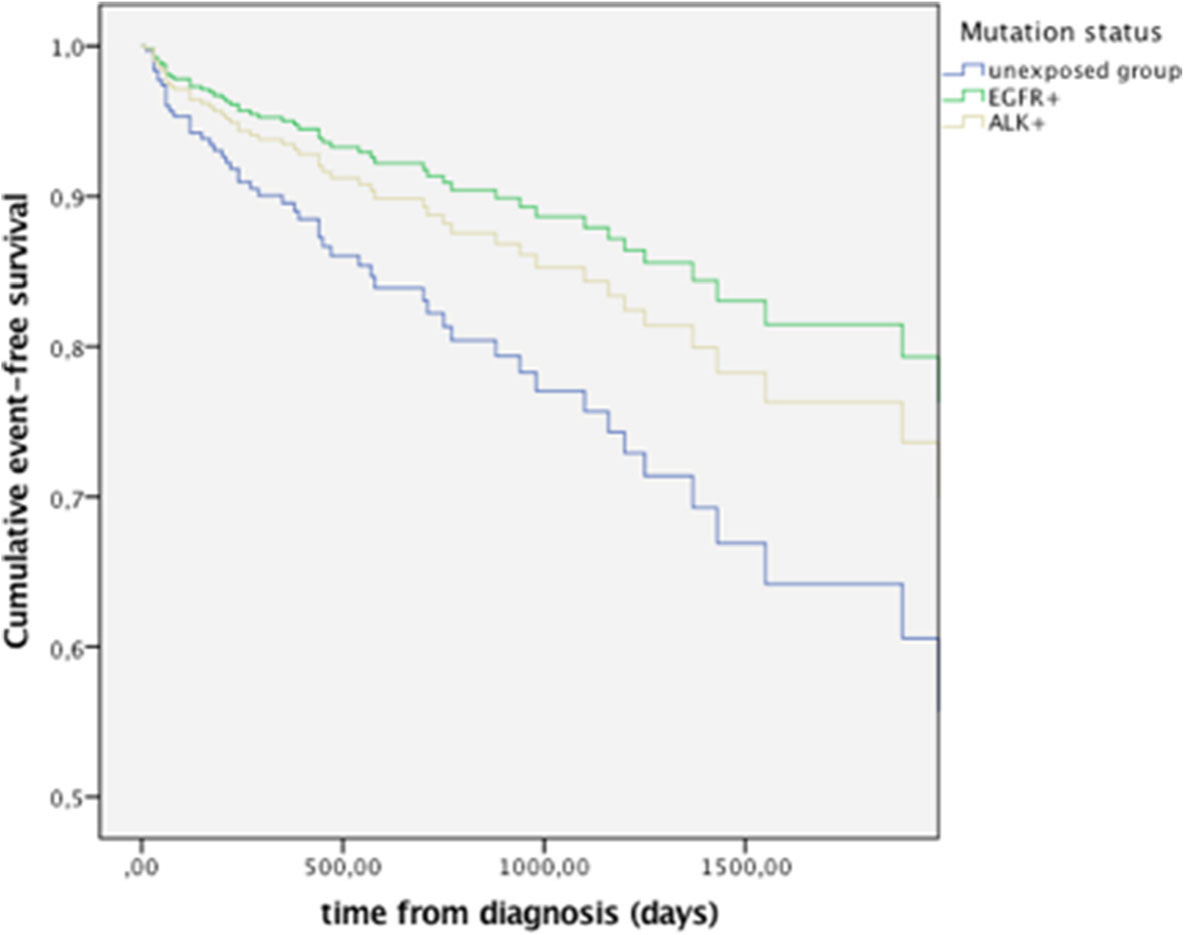
Event-free survival of patients in the Epidermal Growth Factor Receptor (EGFR), anaplastic lymphoma kinase (ALK) mutated groups and in the unexposed group. Calculated with the same Cox regression model as used in Table 3. Demonstrates the difference in event-free survival between the unexposed and mutated groups. Cumulative event-free survival variable: time before the first thromboembolic event

Weighed Cox regression analysis was used to calculate the event-free time for DVT/VTE for the three groups according to mutational status. The unexposed group had a significantly shorter time to first event compared to mutated groups (Fig. 1).

Another Cox regression analysis considering the role of treatments confirmed the protective role of age > 66 and the increased risk of target events in patients with disease in stage IV; all other treatment strategies compared with the reference group chemotherapy alone seemed to exert a protective effect on the risk of target events (Table 5).

**Table 5:** Weighted cox regression analysis for variables affecting event-free time

Variable	HR (95% CI)
Gender	
Male	1
Female	0.88 (0.50–1.55)
Age	
< 66	1
> 66	*0.37 (0.20–0.70)*
Stadium	
I	1
II	2.64 (0.73–9.57)
III	2.40 (0.70–8.24)
IV	*3.63 (1.05–12.50)*
AC before the target event	
NO	1
YES	1,03 (0.45–2.33)
Treatment options	
CTP alone	1
Other treatment without TKI	*0.20 (0.08–0.51)*
Other treatment with TKI	*0.25 (0.12–0.51)*
TKI alone	*0.14 (0.05–0.43)*

*HR* hazard ratio, *95% CI* 95% confidence interval, *NS* not significant, *AC* anticoagulants, preventive dose for venous thromboembolism, *CTP* chemotherapy, *TKI* tyrosine kinase inhibitors

**Fig. 2:**
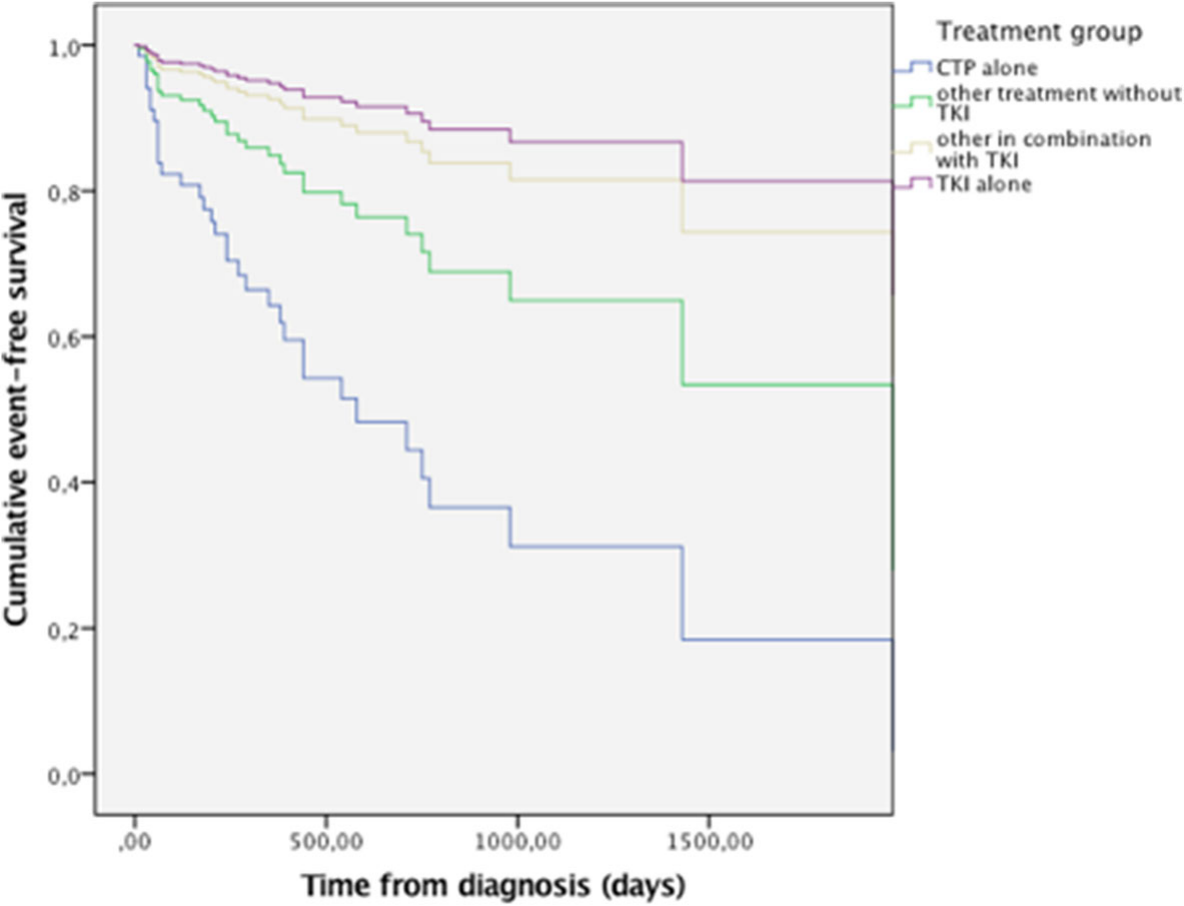
Sensitivity analysis considering risk of thromboembolism for treatments received by patients with lung adenocarcinoma in stage IV during the study period. Calculated with a Cox regression model including only patients with lung adenocarcinoma in stage IV and considering chemotherapy alone as reference for the analysis. Demonstrates the difference in event-free survival between patients treated with tyrosine kinase inhibitors (TKI) alone or in combination with other treatments compared to other treatments not including TKI. CPT: chemotherapy; TKI: tyrosine kinase inhibitors. Cumulative event-free survival variable: time before the first thromboembolic event

A sensitivity analysis was performed considering only patients with disease in stage IV: treatment with TKI alone (HR 0.12 95% CI: 0.04–0.42) or in association with other treatments (HR 0.17 95% CI: 0.07–0.43) clearly resulted in a protective effect for target events when compared to chemotherapy alone, whilst no protective effect was noted for other approaches including chemotherapy. Age > 66 was still protective (HR 0.38 95% CI: 0.18–0.83). Within this group of patients in stage IV, patients receiving chemotherapy alone or in combination with treatments other than TKI had a significantly shorter time to first event compared to patients treated with TKI during the clinical course of their disease (Fig. 2).

## Discussion

The purpose of this study was to investigate if mutational status affected the risk of thromboembolism in patients with lung adenocarcinoma. The main outcome was that patients bearing a EGFR mutation had a lower risk of target events. This result was not confirmed for patients bearing a ALK rearrangement, but this was mainly due to the limited sample size of this group of patients (a trend for protection was observed in the main analysis reported in Table 4).

Treatment with TKI also appeared to further decrease the risk of thromboembolic events, both when used as the only treatment or in combination/sequence with other treatments during the course of the disease. Most mutated patients received TKI, which makes it difficult to fully distinguish the protective effect of the treatment itself.

Mutational status is independent of stage, whilst treatment with TKI is related to stage; this is why the protective effect of mutation is a more consistent finding. The result regarding TKI treatment is partially related to mutational status but is also due to the complexity of clinical management of lung cancer patients. Our study included patients who received both TKI and chemotherapy in different phases of the disease, as well as patients treated with only chemotherapy or chemotherapy in combination with treatments other than TKI. A potential limit is represented by the fact that the exact duration of TKI treatment or of other forms of treatment was not assessed in this study. Nevertheless, the unexposed group not only had an increased risk of target events, but also the time to the first event event was shorter.

According to a preclinical study [21] on colorectal cancer cells, inhibition of EGFR decreases the expression of tissue factor; this would offer a stimulating hypothesis to explain our results; studies investigating specifically which tissue factors are affected in vivo by different treatments might result in the discovery and validation of biomarkers for treatment response and prediction of comorbidities like DVT/VTE.

A previous study by Lee et al. [22] investigated risk factors of thromboembolism in non-small cell lung cancer patients. No significant differences were found between mutated and non-mutated patients. Their study had several flaws for this purpose, e.g., not all patients were tested for mutations and the histology was not specifically adenocarcinoma. They did find an increased risk associated with TKI treatment and hypothesized that this could partly be explained by extensive chemotherapy treatment and longer survival in EGFR-mutated patients. Another study by Yang et al. [23] showed an increased DVT/VTE risk with TKI treatment. The aim of this study was to evaluate the effect of postoperative treatment on DVT/VTE on all types of lung cancer, and neither clinical stage nor other treatments were taken into account when analyzing the increased risk of target events with TKI. Those who received TKI in this study were in a more advanced stage and received extensive treatment, which likely affected the results. In addition, only 32 out of 1001 patients received TKI.

The finding that chemotherapy could increase the risk of DVT/VTE is in accordance with previous studies [15, 18, 22, 24], as well as the risk of DVT/VTE associated with advanced clinical stage [15, 24]. The higher occurrence of thromboembolic events in our study compared to others [17–19] could depend on the fact that data were analyzed from a highly selected group of patients affected by lung adenocarcinoma. This group is known to have higher risk of DVT/VTE compared to other histotypes [17–19, 22]. Furthermore, treatment with cisplatin-based regimens is a strong risk factor for DVT/VTE in patients with non-small cell lung cancer [25]; the vast majority of the patients included in this study were treated with cisplatin-based regimens, as these treatments are still the first choice in patients with advanced adenocarcinoma not bearing a EGFR- or ALK-mutation according to the current Swedish guidelines [11]. Another possibility is that mutated patients are more often diagnosed at an advanced stage; the follow up period was also longer compared to previous studies.

This was a retrospective study in which selection of patients was based on a list of mutational analyses obtained from the Pathology Department of the Karolinska University Hospital. This can raise the concern about a potential selection bias, as we did not enroll prospectively all the patients with lung adenocarcinoma diagnosed in the catchment area. Furthermore, we cannot exclude that not all the patients diagnosed with lung adenocarcinoma during the study period underwent molecular testing. Nevertheless, the proportion of EGFR and ALK mutations in the cohort used to select our study population is consistent with data reported in other series [26], and this should reinforce the validity of our findings.

A major strength of this study compared with previous studies [22, 23] on the risk of thromboembolism in patients with lung cancer is that it did include only patients with lung adenocarcinoma and that patients were specifically selected on the base of the results of the mutational analysis. In particular, the patients included in the unexposed group were truly negative at the molecular testing, confirming that they in fact did not bear any mutation. Previous studies [22, 23] have not found such a correlation but they included non-selected/non-tested populations.

In our study, older patients (with age > 66 years) showed a decreased risk of thromboembolism. One reason could be that younger patients are treated more aggressively, with higher doses of chemotherapy. Another plausible explanation could be that the cancer itself is more aggressive in younger patients. The fact that age > 66 is protective for DVT/VTE seems to contradict general risk factors for thromboembolism [15, 27], but previous studies support our finding [17, 19]. This study did not get any significant results on association between performance status and VTE/DVT and previous studies have been inconclusive [28, 29].

A major limitation of this study was the sample size, especially for the ALK group. Another limitation of our design is that variables such as performance status, smoking status and clinical stage have been treated as if they were constant from diagnosis, when they can vary over time; furthermore, the study was designed to evaluate the risk of DVT/VTE in relation to mutation status and not specifically in association with treatment and variation of treatment over time; nevertheless, our results show that the treatment prescribed greatly influence the risk of target events, and this should be taken in consideration when managing the patients with higher risk.

## Conclusions

Our study shows for the first time that there is a strong correlation between the risk of severe clinical complications and mutational status in lung adenocarcinoma, and that targeted therapies have a safer profile than systemic chemotherapy even for events not directly connected to cancer growth as thromboembolism; this study reinforces the concept that precision medicine is preferable in lung cancer [2]. Furthermore, international guidelines have so far failed to provide strong recommendation for the use of anticoagulants as prophylaxis of DVT/VTE in lung cancer (16); the results of our study can help to identify which patients with lung adenocarcinoma would benefit the most from such interventions.

## Abbreviations

95% CI: 95% confidence interval
ALK: Anaplastic lymphoma kinase
ATP: Adenosine triphosphate
CTP: Chemotherapy
DVT: Deep venous thrombosis
EGFR: Epidermal growth factor receptor
FISH: Fluorescence *in situ* hybridization
HR: Hazard ratio
RTP: Radiotherapy
SD: Standard deviation of the mean
TKI: Tyrosine kinase inhibitors
TNM: TNM classification of malignant tumours
VTE: Venous thromboembolism
